# Predictors of first-line antiretroviral therapy failure among adults and adolescents living with HIV/AIDS in a large prevention and treatment program in Nigeria

**DOI:** 10.1186/s12981-020-00317-9

**Published:** 2020-11-03

**Authors:** Nicaise Ndembi, Fati Murtala-Ibrahim, Monday Tola, Jibreel Jumare, Ahmad Aliyu, Peter Alabi, Charles Mensah, Alash’le Abimiku, Miguel E. Quiñones-Mateu, Trevor A. Crowell, Soo-Yon Rhee, Robert W. Shafer, Ravindra Gupta, William Blattner, Manhattan E. Charurat, Patrick Dakum

**Affiliations:** 1 Institute of Human Virology, Federal Capital Territory, 252 Herbert Macaulay Way, Abuja, Nigeria; 2 Institute of Human Virology, University of Maryland School of Medicine, Baltimore, MD USA; 3 University of Abuja Teaching Hospital, Federal Capital Territory, Abuja, Nigeria; 4 Department of Microbiology and Immunology, University of Otago, Dunedin, New Zealand; 5 Henry M. Jackson Foundation for the Advancement of Military Medicine, Bethesda, MD USA; 6 U.S. Military HIV Research Program, Walter Reed Army Institute of Research, Silver Spring, MD USA; 7 Department of Medicine, Stanford University, Stanford, CA USA; 8 University College London, London, UK

## Abstract

**Background:**

A substantial number of persons living with HIV (PLWH) in Nigeria do not experience durable viral suppression on first-line antiretroviral therapy (ART). Understanding risk factors for first-line treatment failure informs patient monitoring practices and distribution of limited resources for second-line regimens. We determined predictors of immunologic and virologic failures in a large ART delivery program in Abuja, Nigeria.

**Methods:**

A retrospective cohort study was conducted at the University of Abuja Teaching Hospital, a tertiary health care facility, using data from February 2005 to December 2014 in Abuja, Nigeria. All PLWH aged ≥ 15 years who initiated ART with at least 6-month follow-up and one CD4 measurement were included. Immunologic failure was defined as a CD4 decrease to or below pre-ART level or persistent CD4 < 100 cells per mm^3^ after 6 months on ART. Virologic failure (VF) was defined as two consecutive HIV-1 RNA levels > 1000 copies/mL after at least 6 months of ART and enhanced adherence counselling. HIV drug resistance (Sanger sequences) was analyzed using the Stanford HIV database algorithm and scored for resistance to common nucleoside reverse transcriptase inhibitors (NRTIs) and non-nucleoside reverse transcriptase inhibitors (NNRTIs). Univariate and multivariate log binomial regression models were used to estimate relative risks (RRs) and 95% confidence intervals (CIs).

**Results:**

Of 12,452 patients followed, a total of 5928 initiated ART with at least 6 months of follow-up and one CD4 measurement. The entry point for 3924 (66.2%) was through the program’s own voluntary counseling and testing (VCT) center, while 1310 (22.1%) were referred from an outside clinic/program, 332 (5.6%) in-patients, and 373 (6.3%) through other entry points including prevention of mother to child transmission (PMTCT) and transferred from other programs. The mean CD4 at enrollment in care was 268 ± 23.7 cells per mm^3^, and the mean HIV-1 RNA was 3.3 ± 1.3.log_10_ copies/mL. A total of 3468 (80.5%) received nevirapine (NVP) and 2260 (19.5%) received efavirenz (EFV)—based regimens. A total of 2140 (36.1%) received tenofovir (TDF); 2662 (44.9%) zidovudine (AZT); and 1126 (19.0%) stavudine (d4T). Among those receiving TDF, 45.0% also received emtricitabine (FTC). In a multivariate model, immunologic failure was more common among PLWH with female gender as compared to male [RR (95% CI) 1.22 (1.07–1.40)] and less common among those who entered care at the program’s VCT center as compared to other entry points [0.79 (0.64–0.91)], WHO stage 3/4 as compared to 1/2 [0.19 (0.16–0.22)], or CD4 200 + cells per mm^3^ as compared to lower [0.19 (0.16–0.22)]. Virologic failure was more common among PLWH who entered care at the program’s VCT center as compared to other entry points [RR (95% CI) 1.45 (1.11–1.91) and those with CD4 < 200 cells per mm^3^ at entry into care as compared to higher [1.71 (1.36–2.16)]. Of 198 patient-derived samples sequenced during virologic failure, 42 (21%) were wild-type; 145 (73%) carried NNRTI drug resistance mutations; 151 (76.3%) M184I/V; 29 (14.6%) had ≥ 3 TAMs, and 37 (18.7%) had K65R, of whom all were on TDF-containing first-line regimens.

**Conclusions:**

In this cohort of Nigerian PLWH followed for a period of 9 years, immunologic criteria poorly predicted virologic failure. Furthermore, a subset of samples showed that patients failing ART for extended periods of time had HIV-1 strains harboring drug resistance mutations.

## Background

In 2014, the Joint United Nations Programme on HIV/AIDS (UNAIDS) released the ambitious 95–95–95 targets to end the HIV epidemic globally by 2030, which included mandates that 95% of all diagnosed persons living with HIV (PLWH) should be on antiretroviral therapy (ART) and 95% of those should have plasma HIV-1 RNA suppression [1]. The Government of Nigeria, with support from the US President’s Emergency Plan for AIDS Relief (PEPFAR), The Global Fund to Fight AIDS, Tuberculosis and Malaria and other international and domestic donors, has scaled up ART services in Nigeria in an effort to achieve these targets [2]. From 2006 to 2009, the number of Nigerian PLWH initiated on ART increased from 90,008 [3] to an estimated 300,000 [4]. At the end of 2014, a total of 747,382 or 50% of the estimated 1.5 million PLWH in need of ART were receiving therapy in Nigeria [5].

The Institute of Human Virology Nigeria (IHVN), a PEPFAR implementing partner since 2005, has been cumulatively responsible for screening over 6.6 million people and providing treatment to over 240,000 PLWH. In PEPFAR 2.0, IHVN accounted for 19% of all HIV testing services, 19% of patients receiving treatment, and 20% of women receiving prevention of mother-to-child transmission (PMTCT) prophylaxis in Nigeria. In addition to its 1455 treatment and PMTCT sites, IHVN supported the redistribution of its sites to new implementing partners during the US Government (USG) rationalization policy. IHVN is at the forefront of quality laboratory services through its regional training centers, international certification initiative and in establishment of a subnational viral load and early infant diagnosis (EID) network.

Nigeria adopted in 2015 a ‘test and treat’ policy of ART initiation in all PLWH regardless of CD4, clinical stage, age or population [6]. Although additional work is needed to completely achieve this goal, efforts have been made to scale-up treatment access and the number of PLWH receiving ART almost doubled between 2015 and 2018 [7]. The vast majority of PLWH initiating ART are started on TDF + 3TC + DTG as the preferred first line ART regimen with efforts to maintain this regimen for as long as possible to minimize the costs, toxicities, and inconveniences of second-line regimens [8].

Unfortunately, emergence of resistance to tenofovir disoproxyl fumarate (TDF), a key component of regimens for people starting HIV treatment, has been a particular concern that threatens the efficacy of first-line TDF-containing regimens [9], particularly in those with prior exposure to thymidine analogues before initiation of TDF-containing regimens [10]. Viral load (VL) monitoring is associated with lower prevalence of drug resistance in low and middle income countries (LMIC) [11] and is recommended as the preferred strategy for monitoring ART effectiveness [12]. The main goal is to not only reduce viral failure (VF) and resistance in those on ART, but also to reduce the community-level burden of pre-treatment drug resistance (PDR), which has been rising sharply in sub-Saharan countries [13]. In this study we investigated factors associated with virologic and immunologic failures among adult patients attending a large clinic in Abuja, Nigeria, and characterized HIV-1 drug resistance in this population.

## Materials and methods

### Study design and population

**Fig. 1:**
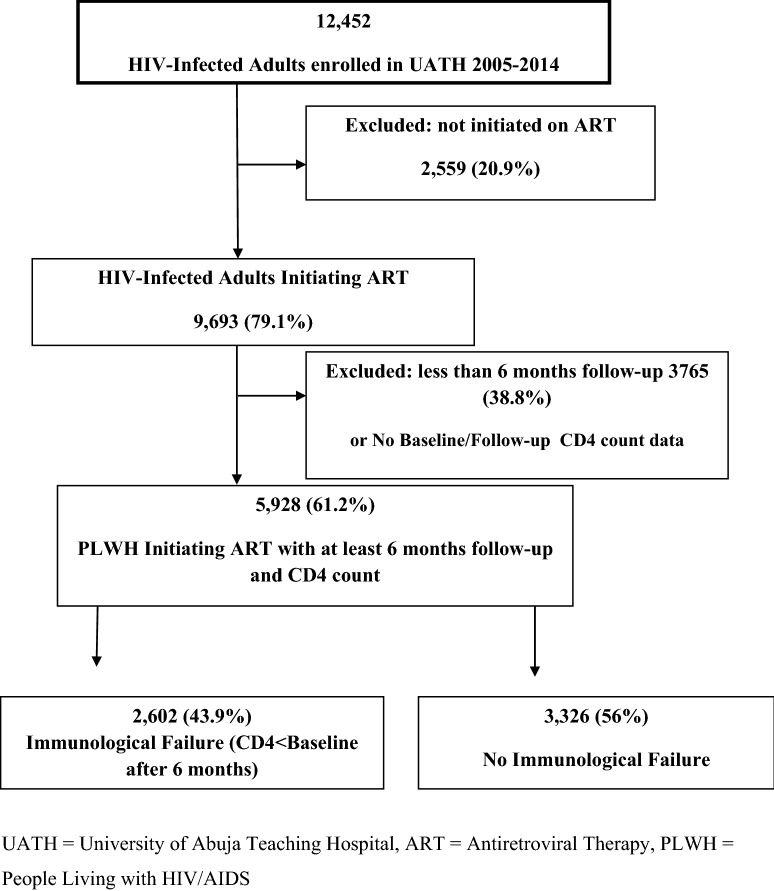
Flowchart highlighting enrolled patient population. *UATH* University of Abuja Teaching Hospital, *ART* antiretroviral therapy, *PLWH* People living with HIV/AIDS

This was a retrospective cohort study using data from adult and adolescent PLWH enrolled in a large ART program funded by PEPFAR at the University of Abuja Teaching Hospital (UATH), a tertiary health care facility supported by IHVN in the North Central region of Nigeria. A total of 12,456 PLWH were enrolled into HIV care from February 2005 through December 2014. Participants included in the study were aged ≥ 15 years at enrollment with a minimum of 6 months on ART, CD4 measurements at entry into care, and a viral load test at least 6 months after ART initiation (targeted viral load monitoring until 2013) (Fig. 1).

Between 2003 and 2010, PLWH at World Health Organization (WHO) stage 4, WHO stage 3 with CD4 ≤ 350 cells/mm^3^ and those with CD4 ≤ 200 cells/mm^3^ irrespective of WHO staging were considered eligible for ART. After 2010, all PLWH with CD4 ≤ 350 cells/mm^3^ irrespective of WHO staging and those at WHO Clinical stage 3 or 4 were eligible for ART [14].

### Clinical procedures and data collection

ART eligibility was based on the Nigerian National guidelines for HIV and AIDS treatment and care among adolescents and adults. At the beginning of program implementation, the most common first-line ART included stavudine (d4T), lamivudine (3TC), and nevirapine (NVP). In late 2006, the increased recognition of the toxicity and inferior efficacy of regimens containing d4T prompted the revision of international guidelines, with eventual removal of d4T from recommended first-line regimens. In 2008–2009, the introduction of generic tenofovir (TDF) equivalents and the fixed-dose combination (FDC) with emtricitabine (FTC) and efavirenz (EFV) further expanded usage of TDF in lieu of d4T [9, 15].

PLWH were identified and enrolled in care either through external referrals from other clinics and voluntary counselling testing (VCT) or from internal referrals, from the HIV Testing Services (HTS) center, inpatient hospital, or other clinics within the facility. At clinic enrollment, patients were interviewed by clinic staff to obtain complete intake records. Medical and social histories were obtained, physical examinations were performed, and blood was taken for laboratory evaluations such as CD4 assessment, viral load quantification, and hepatitis B screening. Psychosocial and drugs adherence counseling was performed by trained staff. Adherence was assessed by self-report at each visit.

Clinicians decided whether or not to initiate ART for individual PLWH in consideration of local guidelines. PLWH who did not meet CD4-based criteria for ART initiation at the time of their enrollment into care were scheduled for a follow-up visit 6 months later for another review. Those with CD4 counts that qualified for ART initiation were either started on ART immediately or referred for intensive treatment preparation before ART initiation, depending on guidelines in effect at the time. After ART initiation, patients were scheduled for follow-up visits 2–4 weeks later and then every 2 to 3 months thereafter at the discretion of the physician. At each follow-up visit, patients underwent a history, physical and relevant laboratory investigations and these processes were documented on standardized clinical care cards or for routine clinical care management. Specific clinical and laboratory data from these forms were entered by research assistants into CAREWare (JPROG, New Orleans, LA, USA), an electronic medical record system adapted at supported site and exported into SAS for statistical analysis.

### Laboratory analysis

All laboratory tests were performed on-site at IHVN Asokoro Reference Laboratory designated by the Federal Ministry of Health (FMoH) to anchor the National HIV drug resistance in Nigeria, and one of the African Society for Laboratory Medicine collaborating centers in Africa. During the study period, CD4 cell counts were the mainstay for laboratory monitoring in Nigeria [2]. Patients on ART were expected to receive 6-monthly CD4 counts to evaluate progress on ART. CD4 count measurement was performed using laser-based CD4 T-lymphocyte enumeration (Cyflow SL, Partec, Munster, Germany).

Before 2010, VL quantification was performed using the Roche Amplicor 1.5 (Cobas Amplicor; Roche Diagnostics, Switzerland) with a detection limit of 400 copies/mL. Subsequently, from 2011 to 2014, VL was performed using the Roche Cobas AmpliPrep TaqMan (Cobas Amplicor; Roche Diagnostics, Basel, Switzerland) with a detection limit of 20 copies/mL [16]. Targeted viral load (VL) approach was implemented and second-line therapy was not yet widely available at the time of this study.

### ART failure

Immunologic failure was defined as having a decrease in CD4 count to below the preART level or persistent CD4 < 100 cells per mm^3^ after 6 months on ART. Virologic failure was defined as 2 consecutive VL > 1000 copies/mL after at least 6 months of ART and intensive adherence counselling [17]. Adherence was assessed by self-report.

### HIV-1 genotyping

The cDNA HIV *pol* sequences from a subset of plasma samples (n = 198) from patients with a viral load ≥ 1000 copies/ml were reverse transcribed from viral RNA extracted from previously-unthawed cryopreserved plasma, with Sanger sequencing as described previously [18, 19].

Sequencing electropherograms were visually inspected using Sequencher 5.4 (Gene Codes Corp., Ann Arbor, Michigan, USA) at two independent laboratories to verify that each nucleotide base was covered at least by 3 reads, one of which had to be in the opposite direction as the other two. Multiple sequence alignment was performed using MAFFT v7 and manually curated using Se-Al. A combination of tools such as the HIV-1 Genotyping Tool at the National Center for Biotechnology information, Jumping Profile HMM Tool at GLOBICS, REGA HIV Subtyping Tool at BIOAFRICA, and Maximum Likelihood phylogenetic analysis were used for HIV subtyping. If the HIV-1 subtype(s) for a particular sequence was similar among all tools used, a final subtype result was assigned. If the results were different, neighbor joining phylogenetic trees with relevant HIV-1 reference subtype and circulating recombinant form (CRF) sequences were constructed at various breakpoints and over the span of the whole sequence to determine the genetic relatedness of the sample to reference sequences [19].

### Statistical methods

Study participant characteristics at enrollment were reported as percentages or means with standard deviations (SDs). The percentage of participants experiencing immunologic and virologic failure was calculated among all patients enrolled in the treatment program at UATH. Univariate and multivariate analyses were performed using log binomial models to estimate relative risks (RRs) and 95% confidence intervals (CIs) for associations of factors of interest with immunologic and virologic failure. All available plausible predictors were included in the multivariate models if they had a p value of < 0.20 in univariate analysis. The criterion for significance for all analyses was a 2-sided P value of < 0.05. All statistical analyses were performed using SAS version 9.3 (SAS Institute Inc, Cary, North Carolina).

## Results

### Cohort characteristics

Between February 2005 and December 2014, 12,452 PLWH were followed at UATH. Of these, 5928 initiated ART, had at least a 6-month follow-up visit, at least one CD4 after ART initiation, and were included in the analyses, including 3788 (64%) females with a mean age (SD) of 34.5 (8.6) years. A total of 3610 (61%) patients were married, 3569 (67%) had at least a secondary education and 2075 (35%) were employed. The mean (SD) weight was 59.6 (17.5) kg and body mass index (BMI) was 23.8 (7.0) kg/m^2^ and 1126 (19%) had BMI < 18.5 kg/m^2^. The entry point or mode of enrollment for 3924 (66%) patients was through the UATH VCT program, 1310 (22.1%) from referral by an outside clinic/program, 332 (5.6%) were in-patients and 308 (5.2%) were enrolled through other entry points including prevention of mother to child transmission (PMTCT). Mean (SD) CD4 was 268 (237) cells/mm^3^, and 3058 (52%) patients had CD4 < 200 cells/mm^3^ while 5221 (88%) individuals had CD4 < 500 cells/mm^3^. The mean (SD) VL was 3.3 (1.3) log_10_copies/ml. Demographic characteristics at the time of ART initiation are shown in Table 1.

**Table 1:** Demographic and clinical characteristics of PLWH at enrollment into the University of Abuja Teaching Hospital HIV Treatment Program

Characteristics	N = 5928
Gender, n (%)	
Male	36.1
Female	63.9
Age, mean ± SD years	34.5 ± 8.6
Age, n (%)	
15–24 years	8.7
25–30 years	23.0
30–34 years	23.3
35–39 years	19.6
40–44 years	11.9
45–49 years	7.4
50+ years	6.0
Service entry point, n (%)	
In-patient	5.6
Outside clinic/program	22.1
PMTCT	1.7
VCT	66.2
Other	3.5
ART regimen, n (%)	
D4T, 3TC, NVP	17.7
D4T, 3TC, EFV	1.3
AZT, 3TC, NVP	40.8
AZT, 3TC, EFV	1.9
TDF, XTC, NVP	22.0
TDF, XTC, EFV	14.1
Others	2.1
WHO stage, n (%)	
1	39.7
2	24.4
3	32.0
4	3.9
Weight, mean ± SD kg	59.6 ± 17.5
BMI, mean ± SD kg/m^2^	23.8 ± 7.0
BMI, n (%)	
< 17 kg/m^2^	12.1
17–18.4 kg/m^2^	7.3
18.5–24.9 kg/m^2^	49.7
25–29.9 kg/m^2^	20.8
30+ kg/m^2^	10.1
CD4 count, mean ± SD cells/mm^3^	268 ± 23.7
CD4 Count, n (%)	
< 200 cells/µL	52.2
200+ cells/µL	88.2
log_10_ viral load, mean ± SD copies/mL	3.3 ± 1.3

*PMTCT* prevention of mother-to-child transmission, *VCT* voluntary counselling and testing, *Mean* ± *SD* mean and standard deviation, *n*(*%*) number and percentage, *D4T* Stavudine, *3TC* Lamivudine, *NVP* nevirapine, *EFV* efavirenz, *AZT* zidovudine, *TDF* tenofovir, *XTC* emtricitabine, *ART* antiretroviral therapy, *WHO* World Health Organisation, *BMI* body mass index

### Predictors of immunologic failure

In a multivariate model, immunologic failure was more common amongst patients receiving NVP–based regimens [RR (95% CI) 1.21 (0.99–1.45)] and of female gender [RR (95% CI) 1.22 (1.07–1.40)]. Immunologic failure was less likely among patients identified and enrolled either through VCT [RR (95% CI) 0.79 (0.64–0.91)], at WHO stage 1 or 2 [RR (95% CI) 0.76 (0.60–0.96)] and CD4 < 200, cells/mm^3^ [RR (95% CI) 0.19 (0.16–0.22)] (Table 2).

**Table 2:** Univariate and multivariate analysis of predictors of immunologic failure

Characteristics	Unadjusted RR PR (95% CI)	p-value^1^	Adjusted PRR (95% CI)	p-value^2^
Gender				
Male	1.00		1.00	
Female	0.98 (0.88–1.09)	0.17	1.22 (1.07–1.40)	0.005
Occupation				
Employed	0.92 (0.80–1.05)	0.19	1.18 (0.95–1.46)	0.14
Others	1.00		1.00	
Service entry point				
Voluntary counseling and testing	0.78 (0.70–0.88)	< 0.001	0.79 (0.64–0.91)	0.002
Others	1.00		1.00	
NVP containing regimen				
Yes	1.35 (1.18–1.54)	< 0.001	1.21 (0.99–1.45)	0.23
No	1.00		1.00	
WHO stage				
1 or 2	1.00	0.14	1.00	0.013
3 or 4	0.97 (0.84–1.19)		0.76 (0.60–0.96)	
CD4 cell count, cells/mm^3^				
200 +	0.22 (0.20–0.24)		0.19 (0.16–0.22)	
< 200	1.00	< 0.001	1.00	< 0.001

^1^Prevalence RatioRelative risk (PRRR), 95% confidence interval (CI) and p-values were calculated using log binomial regression

^2^Multivariate models included all predictors significant at p value < 0.20

### Predictors of virologic failure

Factors that were independently associated with virologic failure in a multivariable model included CD4 < 200 cells/mm^3^ at ART initiation [1.71 (1.36–2.16)] and HIV care enrollment via UATH VCT [RR (95% CI): 1.45 (1.11–1.91)] versus other service entry points (Table 3).

**Table 3:** Univariate and multivariate analysis of predictors of virological failure

Characteristics	Unadjusted RR PR (95% CI)	p-value^1^	Adjusted PRR (95% CI)	p-value^2^
Age categories				
< 30	1.00		1.00	
30–39	0.57 (0.42–0.77)	< 0.001	0.45 (0.31–0.67)	< 0.001
40–49	0.56 (0.41–0.76)	< 0.001	0.42 (0.28–0.63)	< 0.001
50 +	0.37 (0.26–0.54)	< 0.001	0.23 (0.14–0.39)	< 0.001
Marital status				
Married	0.73 (0.60–0.91)	0.004	0.71 (0.55–0.97)	0.021
Others	1.00		.00	
Service entry point				
Voluntary counseling and testing	1.61 (1.25–2.07)	< 0.001	1.45 (1.11–1.91)	0.007
Others	1.00		1.00	
WHO stage				
1 or 2	1.00	< 0.001	1.00	0.003
3 or 4	0.52 (0.41–0.74)		0.63 (0.46–0.85)	
CD4 cell count < 200, cells/mm^3^				
< 200	1.43 (1.16–1.76)	< 0.001	1.71 (1.36–2.16)	< 0.001
200 +	1.00		1.00	

^1^ Relative riskPrevalence Ratio (PRR), 95% confidence interval (CI) and p-values were calculated using log binomial regression

^2^ Multivariate models included all predictors significant at p value < 0.20

### Antiviral drug–resistance mutations and HIV subtypes

**Fig. 2:**
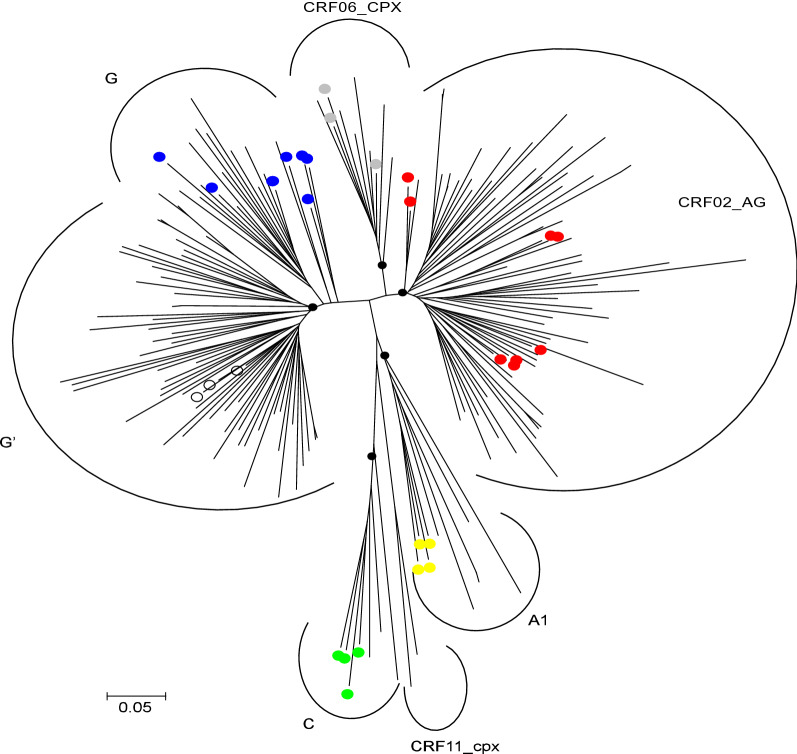
HIV-1 phylogenetic tree was derived from an alignment of pol sequences. HIV-1 reference strains are highlighted

**Fig. 3:**
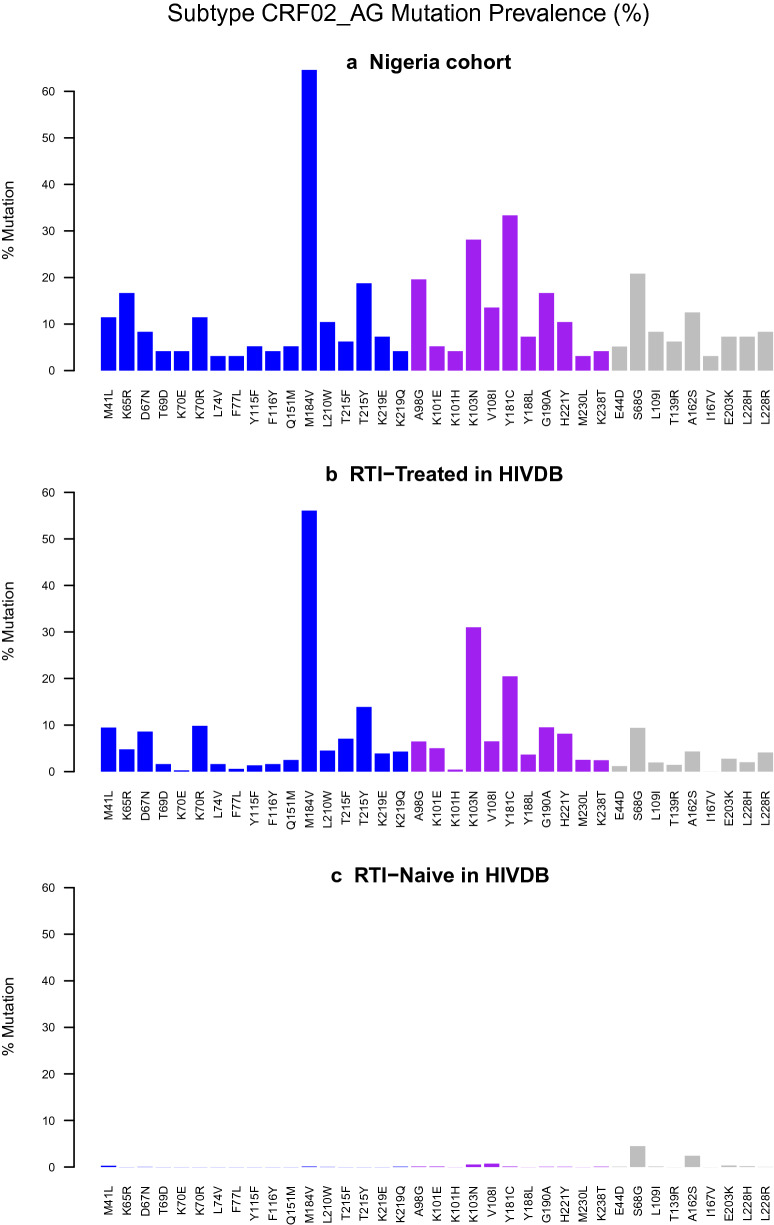
(1) For the 226 mutations, this figure shows its prevalence in CRF02_AG Nigerian patients (**a**), in treated CRF02_AG patients in HIVDB (**b**) and in treatment-naive CRF02_AG patients in HIVDB (**c**). NRTI DRMs are shown in blue, NNRTI DRMs are shown in purple and the remaining mutations are shown in grey. (2) 146 of the 226 mutations occurred ≥ 2 patients in CRF02_AG Nigerian patients. (3) 37 of the 226 mutations occurred significantly more frequently in CRF02_AG Nigerian patients compared to CRF02_AG treatment-naive patients in HIVDB (adjusted Fisher’s exact test p value < 0.01). Those 37 mutations are shown in this figure. *NRTI* nucleoside reverse transcriptase inhibitor, *NNRTI* non-nucleoside reverse transcriptase inhibitor, *DRM* drug resistant mutation, *CRF* circulating recombinant form, *HIVDB* HIV data base, *RTI* reverse transcriptase inhibitor

**Fig. 4:**
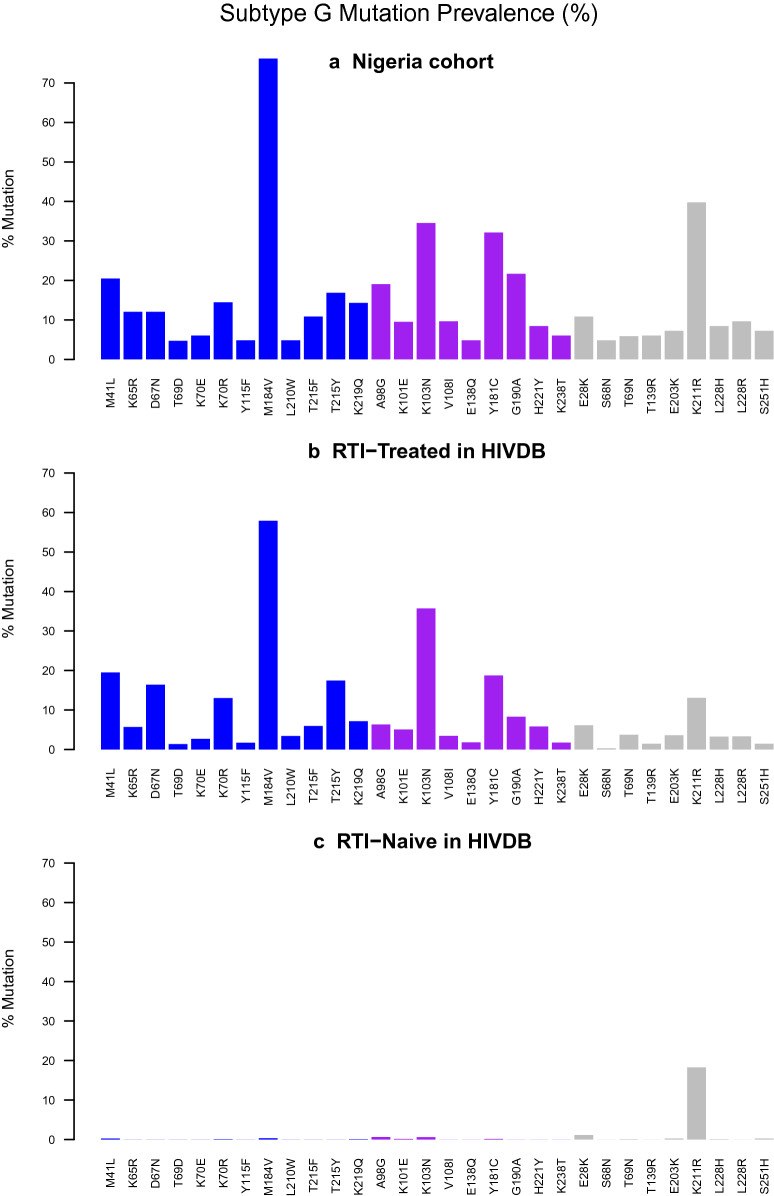
Thirty (n = 30) mutations occurring significantly more frequently in Nigerian subtype G patients compared to treatment-naive subtype G patients in HIVDB are shown in this figure. NRTI DRMs are shown in blue, NNRTI DRMs are shown in purple and the remaining mutations are shown in grey. 30 mutations occurring significantly more frequently in Nigerian subtype G patients compared to treatment-naive subtype G patients in HIVDB shown in this figure

Of the 198 patient-derived HIV-1 sequences obtained from individuals with evidence of virologic failure, CRF02_AG HIV-1 subtype was the most prevalent (n = 96, 48.5%), followed by subtype G (n = 83, 42%) and other subtypes or CRFs (n = 179, 9.5%) (Fig. 2). CRF02_AG viruses harbored a total of 226 RT mutations, defined as amino acid changes relative to CRF02_AG consensus amino acid sequence (Los Alamos HIV Database). For subtype G sequences, there were 227 RT mutations compared to subtype G consensus amino acid sequence (Los Alamos HIV Database). For each of the 226 mutations present in 96 CRF02_AG sequences, we compared its frequency to the prevalence in treatment-naive subtype CRF02_AG patients in the Stanford HIV Drug Resistance Database (HIVDB). Similar analysis was done with the subtype G sequences. We created three plots for subtype CRF02_AG: For the 226 mutations, Fig. 3 shows its prevalence in CRF02_AG Nigerian samples, ART-experienced CRF02_AG patients in HIVDB and ART -naive CRF02_AG patients in HIVDB. Thirty-seven of the 226 mutations occurred significantly more frequently in CRF02_AG Nigerian samples compared to CRF02_AG treatment-naive samples in HIVDB (adjusted Fisher’s exact test p value < 0.01). Those 37 mutations are shown in Fig. 3 and 30 mutations occurring significantly more frequently in Nigerian subtype G patients compared to treatment-naive subtype G patients in HIVDB shown in Fig. 4.

## Discussion

Our study evaluated immunologic and virologic failure in a large African HIV treatment program. We found that predictors of immunologic failure included female gender, entry into care via VCT, and low CD4. Predictors of virologic failure included young age, unmarried status, entry into care via VCT, WHO stage 3/4, and low CD4. Immunologic criteria poorly predicted virologic failure.

Although some studies have examined predictors of immunologic failure among patients failing first-line regimens, there is a paucity of data from sub-Saharan Africa [20–25]. In a retrospective multi-site study evaluating long-term outcomes of patients receiving ART in a treatment program in Nigeria, authors indicated that older age, ART regimen, lower CD4 count, higher VL and inadequate adherence were predictors of virologic failure, while female sex and lower baseline CD4 were predictive of immunologic failure [14]. Our findings in North Central Nigeria are consistent with that prior work with respect to the impact of gender and age on risk of immunologic failure as well as the impact of age and CD4 at ART initiation on risk of VF.

Many PLWH in sub-Saharan Africa are diagnosed late in the disease course, with low CD4 counts [26]. In this study, more than 50% of participants had CD4 counts < 200 cells/mm^3^ at entry into care. This may be related to patient-level factors such as lack of awareness of their HIV status or barriers to accessing care such as stigma [27, 28], high costs or distance from clinics [29].

Consistent with our findings, data from a large cohort study conducted in East Africa and Nigeria showed that younger age, single marital status and female gender were independently associated with virologic failure and immunologic failure [25]. In addition, at a tertiary hospital such as UATH, the clinicians who manage patients are largely resident doctors who rotate through various departments, including the HIV treatment clinic, with variable supervision. Instability and attrition of trained clinicians may result in suboptimal care that creates additional barriers to engagement and retention of PLWH (Ndembi et al. unpublished data).

Similar to previous studies [18, 30–33], we observed that HIV-infected individuals in this study were predominantly infected with CRF02_AG and subtype G. Although polymorphisms were detected in different proportions among distinct subtypes, the prevalence of a particular HIV-1 variant in one subtype rarely differed by more than tenfold compared with the prevalence of that variant in a different subtype. Interestingly, a significantly higher rate of K65R mutation was observed among patients infected predominantly with CRF02_AG (16.7% of K65R) and G (12.0%) HIV-1 strains on first-line ART in this West African setting. Our findings on subtype variability are similar to those from other published studies of drug resistance in patients with subtypes G and CRF02_AG [34]. Lack of routine VL monitoring limits the early detection of VF, thus promoting the accumulation of drug-resistance-associated mutations [5, 35, 36]. This may account for the high prevalence of NRTI and NNRTI resistance mutations in cohorts from resource-limited settings [9, 11, 14, 37].

Based on the reported high virological responses to d4T-containing first-line ARV regimens, it is expected that the majority of adherent adult patients receiving a first-line d4T-containing regimen will be virologically suppressed at the time of d4T switched. Because of its favorable toxicity profile, TDF will usually be preferable to AZT in these patients. TDF will retain residual activity against emerging drug-resistant variants.

## Conclusion

In this cohort of Nigerian PLWH, there was a high burden of drug resistance among patients failing ART for extended periods of time. Proactive identification of participants at risk for virologic failure is needed to optimize HIV care outcomes and decrease the risk of acquired drug resistance. Immunologic criteria poorly predicted virologic failure, underscoring the need for consistent resources to support routine viral load monitoring in Nigeria and other resource-limited settings. As ART coverage increases globally, HIV drug resistance remains one of the main obstacles to achieving the 95–95–95 treatment targets.

## Data Availability

The data that support the findings of this study are available from the corresponding author, [NN], upon reasonable request.
